# The microbiota and microbiome in pancreatic cancer: more influential than expected

**DOI:** 10.1186/s12943-019-1008-0

**Published:** 2019-05-20

**Authors:** Miao-Yan Wei, Si Shi, Chen Liang, Qing-Cai Meng, Jie Hua, Yi-Yin Zhang, Jiang Liu, Bo Zhang, Jin Xu, Xian-Jun Yu

**Affiliations:** 1 Department of Pancreatic Surgery, Fudan University Shanghai Cancer Center, Shanghai, 200032 China; 2 Department of Oncology, Shanghai Medical College, Fudan University, Shanghai, 200032 China; 3 Pancreatic Cancer Institute, Fudan University, Shanghai, 200032 China; 4 Shanghai Pancreatic Cancer Institute, Shanghai, 200032 China

**Keywords:** Pancreatic cancer, Microbiota, Microbiome, Inflammation, Metabolism, Immunotherapy, Tumor Microenvironment

## Abstract

Microbiota is just beginning to be recognized as an important player in carcinogenesis and the interplay among microbes is greater than expected. Pancreatic ductal adenocarcinoma (PDAC) is a highly lethal disease for which mortality closely parallels incidence. Early detection would provide the best opportunity to increase survival rates. Specific well-studied oral, gastrointestinal, and intrapancreatic microbes and some kinds of hepatotropic viruses and bactibilia may have potential etiological roles in pancreatic carcinogenesis, or modulating individual responses to oncotherapy. Concrete mechanisms mainly involve perpetuating inflammation, regulating the immune system-microbe-tumor axis, affecting metabolism, and altering the tumor microenvironment. The revolutionary technology of omics has generated insight into cancer microbiomes. A better understanding of the microbiota in PDAC might lead to the establishment of screening or early-stage diagnosis methods, implementation of cancer bacteriotherapy, adjustment of therapeutic efficacy even alleviating the adverse effects, creating new opportunities and fostering hope for desperate PDAC patients.

## Background

The ecological community of microorganisms is familiarly known as the microbiota, and developing the discipline of microbiology. The steady state, abundance, and diversity of the microbiota are vital to health. Microbiome, meaning the comprehensive genomic information encoded by the microbiota and its ecosystem, products and host environment, has attracted substantial attention. However, most researchers use “microbiota” and “microbiome” interchangeably. The human microbiota offer protection from disease by supporting nutrition and hormonal homeostasis, modulating inflammation, detoxifying compounds, and providing bacterial metabolites that have metabolic effects [1]. It develops throughout life after birth, and factors including extrinsic modulators and host intrinsic factors may cause its wide microbial diversity [2]. Specifically, extrinsic modulators include diet, antibiotics, drugs, environmental stressors, exercise/lifestyle, gastric surgery. Also, the microbial community presents with highly personalized and interindividual variability, which depends on host specifics such as age, gender, genetics, hormones, and bile acids [3]. Among these factors, host genotype and diet seem to be the most important; furthermore, host and microbial genotypes influence cancer susceptibility. Notably, pancreatic acini can secret mediators that shape the gut microbiota and immunity [4].

Microbiota resides on or within ~ 20% of human malignancies [5]. Recent studies of the impact of the microbiota on carcinogenesis highlighted its crucial roles in gastrointestinal malignancies such as colorectal [6–11], liver [12–16] and pancreatic cancer. Interestingly, the host microbes may increase, decrease, or show no effect on tumor susceptibility [17] and amplify or mitigate carcinogenesis. Pancreatic ductal adenocarcinoma (PDAC) is a lethal and devastating malignancy, as 94% of patients succumb to the disease within 5 years of diagnosis. Acknowledged nonhereditary conditions associated with high PDAC risk include age (> 55 yo), chronic pancreatitis, diabetes, tobacco smoking, obesity, alcohol abuse, dietary factors, and toxin exposure [18]. Radical surgery still affords the only chance of cure for PDAC, and very few treatments are currently available. Early detection would provide the optimum opportunity to improve the survival rate and quality of life in patients, but to date, there are no well-recognized screening tools or biomarkers at the population level.

Given the growing evidence suggesting that microbes are related with PDAC susceptibility, initiation, progression and can influence therapeutic efficacy, while the mechanisms involved are still being deciphered, the role of the microbiota in pancreatic carcinogenesis requires closer attention. This article aims to review recent developments and intriguing discoveries in pancreatic cancer microbiota and microbiome research, to illustrate the underlying mechanisms, and then, to discuss potentially relevant clinical applications and promising future directions.

### Specific microbiota associated with PDAC: the state of the art

**Fig. 1:**
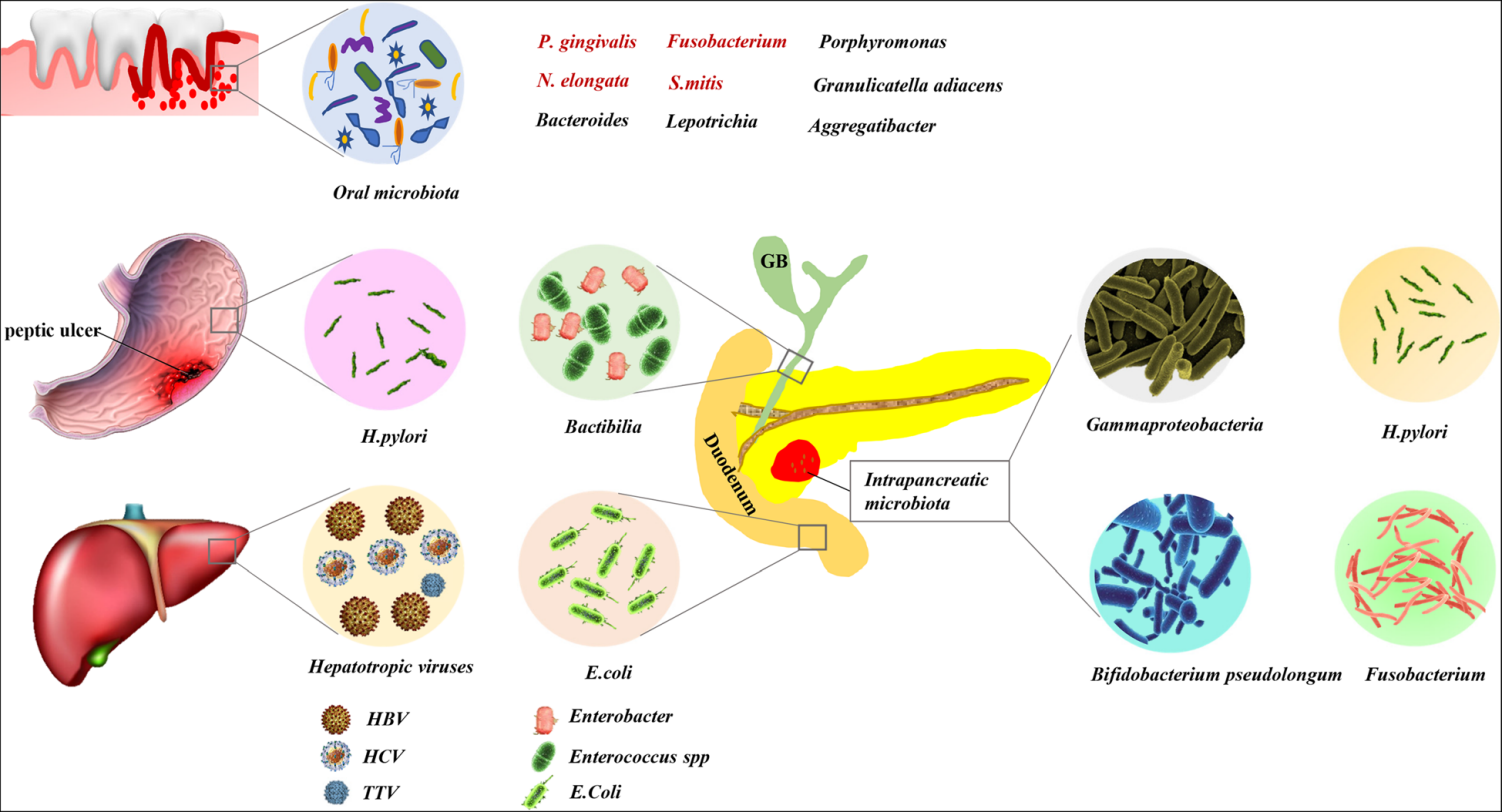
Specific Microbiota Associated with PDAC. *P. gingivalis*, *Fusobacterium*, *N. elongata* and *S. mitis* are keystone pathogens among oral bacteria involved in carcinogenesis. *H. pylori* infection is associated with an increased risk of developing PDAC. The potential oncogenic role of hepatotropic viruses, including HBV/HCV and TTV, in PDAC, although molecular evidence is scarce. Bactibilia, including *Enterobacter* and *Enterococcus* spp., and gut microbes represented by *E. coli* ultimately lead to the development of PDAC. Abundant intratumoral microbes were found in PDAC tissues compared with normal pancreas

Diverse microbiota alterations exist in patients with PDAC compared to healthy groups at several body sites, including oral, GI, and pancreatic tissues [19]. To study these microbiota, scientists serially collected clinical and epidemiological data and examined various microbes in oral mouthwash/swab, salivary, blood, stool, biopsy, and tissue samples. Main detection methods include plasma antibody analysis, 16S ribosomal RNA (16S rRNA) gene sequencing, quantitative polymerase chain reaction (qPCR), microarray and enzyme-linked immunosorbent assay (ELISA). Many studies have found that the oral microbiota, periodontal disease, and tooth loss play pivotal roles in pancreatic carcinogenesis. Epidemiological studies demonstrate that *Helicobacter pylori (H. pylori)* may be a risk factor for PDAC, and the potential oncogenic role of hepatitis B virus *(HBV)* in pancreatic tumorigenesis is supported by clinical observation, although molecular evidence is scarce. Studies on the intrapancreatic microbiota have also been performed. The specific microbiota associated with PDAC are summarized in Fig. 1. Above findings may provide a hypothesis that PDAC may have some bacterial origins.

### Oral microbiota, periodontal disease and tooth loss

Over 700 different microorganism species colonize the human oral cavity, and the oral microbiota [20] remains relatively stable in healthy population. However, most oral microbes have not been cultured in a laboratory [21, 22], which limits research. Periodontitis is a chronic oral inflammation of the gingiva and surrounding tissues [23]. It is the most common infectious condition leading to tooth loss and has been linked with various cancers of the pancreas [24–30], colorectum [27] and other extraintestinal organs [31–35]. Poor oral health status, pathogenic oral flora [36], periodontal disease [24, 25, 27, 37] and tooth loss [37–39] are well-established independent risk factors for PDAC.

Both in animal models and human subjects, microbiologist has verified the spread of oral microbes to pancreas via translocation or dissemination [28, 40]. Moreover, there are parallels to be drawn between the microbiota isolated from the pancreas and oral, especially in cases of pancreatitis [18, 41]. Researchers hold the opinion that oral microbiota dysbiosis preceded the development of PDAC rather than developing after cancer [42]. According to the present literature, *Porphyromonas gingivalis (P. gingivalis)*, *Fusobacterium*, *Neisseria elongata (N. elongata)* and *Streptococcus mitis (S. mitis)* are keystone pathogens among the oral bacteria involved in PDAC carcinogenesis. In the foreseeable future, clinicians may utilize markers in salivary or mouthwash samples for noninvasive, economical screening.

#### P. gingivalis

*P. gingivalis* is a pathogenic bacterium that responsible for chronic periodontitis. Through collecting prediagnosis oral wash samples from participants, Fan et al. [43] characterized the composition of the oral microbiota in samples from 361 people who developed PDAC and 371 healthy participants matched by age, sex, race, body mass index, smoking status, alcohol use and diabetes. This study suggested that *P. gingivalis* was correlated with a 59% greater risk of developing PDAC and played a role in its etiology. Higher levels of ATTC 53978 antibodies against *P. gingivalis* in blood (> 200 ng/mL) were found in 405 patients with PDAC than in healthy volunteers and were related with a 2-fold higher risk of developing PDAC based on a large European cohort [28], which suggests that ATTC 53978 may serve as the best indicator for aggressive periodontal disease and PDAC susceptibility. The above conclusions are consistent with extensive evidence such as the NHANES I [24] and III data [27] and the Health Professionals Follow-up Study [25]. Thus, *P. gingivalis* was the periodontal pathogen most strongly associated with an elevated risk of PDAC.

*P. gingivalis*, *Treponema denticola*, and *Tannerella forsythia*, commonly known as the red complex, are the major periodontitis-causing pathogens; they secrete peptidyl-arginine deiminase (PAD) enzymes and have been extensively studied in patients with PDAC. High mutation rates in the tumor suppressor gene *p53* and oncogene *K-ras*, particularly arginine mutations, known as *p53* Arg72Pro [44, 45] and *K-ras* codon 12 arginine mutations [46–49], respectively, indicates poor prognosis of PDAC patients. These oral bacteria that produce PAD enzymes are capable of degrading arginine, which may result in *p53* and *K-ras* point mutations [50]. Scientists have hypothesized that *P. gingivalis* plays a critical role in initiating inflammation backed by the evidence that individuals with periodontal disease manifest as elevated markers of systemic inflammation and escaping the immune response related to lipopolysaccharide (LPS) and toll-like receptors (TLRs) [51–53].

#### Fusobacterium

Strains of *Fusobacterium* is an anaerobic, gram-negative oral bacterium, which has been verified that could cause periodontal diseases and should always be treated as a pathogen [54]. However, some studies have drawn the opposite conclusions. According to a case-control study [43] and a large prospective cohort study [28], *Fusobacteria* was associated with reduced PDAC risk. Recent studies revealed that *Fusobacterium* potentiates tumorigenesis [55] and promotes chemoresistance in colorectal cancer [56]. More specifically, *Fusobacterium* could increase production of reactive oxygen species (ROS) and inflammatory cytokines, and modulate the tumor immune microenvironment and drive myeloid cell infiltration in intestinal tumors. Despite these conflicting results, the paradoxical effects observed in different tumors may provide evidence to unravel the potential mechanisms.

#### *N. elongata* and *S. mitis*

*N. elongata* and *S. mitis* were found to be lower in saliva specimens collected after PDAC diagnosis than in controls in both the test cohort (10 pairs) and validation dataset (28 pairs) of a retrospective study [36]. This finding was partly supported by Michaud et al. [28], who showed an inverse association of *S. mitis* antibodies with pancreatic cancer (but did not measure *N. elongata*). Farrel et al. [36] found that the combination of *N. elongata* and *S. mitis* biomarkers yielded 96.4% sensitivity and 82.1% specificity in distinguishing patients with PDAC from healthy subjects.

#### Others

The levels of the genera *Corynebacterium* and *Aggregatibacter* are present in lower concentrations in PDAC patients than healthy population, while *Bacteroides* and *Granulicatella adiacens* are more frequent in PDAC salivary RNA samples [36]. However, another study indicated that *Aggregatibacter actinomycetemcomitans* was linked with a higher risk of PDAC [43]. In addition, *Leptotrichia* is considered a protective microbe that dose-dependently decreases the risk of PDAC. Furthermore, a higher ratio of *Leptotrichia* to *Porphyromonas* in saliva can be observed in PDAC patients [57].

### GI microbiota, hepatotropic viruses, and bactibilia

The gut microbiota is a complex and delicate ecosystem of a hundred trillion microbes and the largest microbial community in the human body, protecting bodies from infection, aiding digestion, and regulating gut hormone secretion [58] and the immune system. Disturbances in the gut microbiota could lead to pathology, especially diseases related to metabolism and autoimmunity. Recently, several studies have also shed light on the role of microbiota in carcinogenesis. Among the best studied are the relationships of the gut microbiota to colorectal cancer. Intestinal bacteria are necessary for the breakdown of hydrolytic enzymes secreted by the pancreas; on the other hand, the antibacterial activity of pancreatic juice may protect the pancreas from retrograde infections and contribute to the uniqueness of the intestinal flora. However, gut microbes can reach the pancreas through the circulatory system or the biliary/pancreatic duct (transductal transmission) [59, 60], which may lay the foundation of their potential etiological roles in pancreatic cancer. Depletion of the gut microbiota via oral antibiotics restrained tumor growth and metastatic burden in PDAC mice models, activating antineoplastic immunity in tumor environment simultaneously [61], suggesting that such combinations merit subsequent exploration. Apart from the GI microbiota, represented by *H. pylori*, numerous studies have revealed important associations of HBV and bactibilia with an increased risk of PDAC.

#### *H. pylori*

Researchers are only beginning to explore how *H. pylori* as an engender of PDAC and its role in manipulating the host immune response. Most studies to date, including case-control studies [62–64], prospective cohort studies [65, 66], and meta-analyses [67–69], have confirmed that *H. pylori* infection is related with increased PDAC risk. However, some studies have found no relationship between the two [70–73], and several studies have even drawn the opposite conclusion [74, 75]. One of the difficulties for untangling these inconsistent and paradoxical associations is how to exclude confounding factors.

Using the *H. pylori* IgG antibody level in blood serum from PDAC patients and healthy controls, scientists found that the *H. pylori* IgG level was higher in PDAC. *H. pylori* strains that express Cytotoxin-associated gene A (Cag-A) is associated with gastric inflammation and ulceration and promotes malignant transformation in gastric cancer [76, 77]. Case studies of *H. pylori* antibody-positive in PDAC revealed complicated results related to Cag-A status, and we believe that *H. pylori* and Cag-A predominance in PDAC microbiota studies. One study [78] proposed that factors including ABO blood type may also participate in this intricate process, while a recent meta-analysis revealed a modestly significant increased risk of Cag-A-negative *H. pylori* strain [67–69, 73] with positively correlated factors, including non-O blood type [64, 78] and smoking status [63, 65] in PDAC.

*H. pylori* causes gastric lesions via directly impairing the gastric mucosa, and its DNA can be detected in infected antrum and corpus stomach tissues. However, the expression of *H. pylori* DNA cannot be detected in pancreatic juice or tissues by PCR in chronic pancreatitis and PDAC [79], which may suggest that *H. pylori* cannot trigger pancreatic carcinogenesis directly. Possible indirect mechanisms include inflammation and immune escape. Exposure to carcinogenic nitrosamines is also an underlying mechanism [78]. More specifically, nitrosamine levels are lower in patients with duodenal ulcers than gastric ulcers that characterized by low acidity, which may explain the positive association of PDAC risk with gastric ulcers but not duodenal ulcers [66, 80].

#### *HBV* and Hepatitis C Virus (*HCV*)

*HBV* and *HCV* are hepatotropic viruses that lead to hepatitis and hepatocellular carcinoma (HCC). However, *HBV/HCV* infection is not restricted to the liver; these viruses can be detected in extrahepatic tissues, including the pancreas [81–87], which may play a role in the carcinogenesis or development of extrahepatic malignancies [88–91], including PDAC [85, 86, 89, 91, 92]. Specifically, investigators detected HBsAg and HBcAg in the cytoplasm of pancreatic acinar cells [82], and individuals with chronic *HBV* infection are accompanied by elevated serum and urinary levels of pancreatic enzymes partly [93, 94]. HBsAg was also observed in pancreatic juice among patients with *HBV* infection [95] and was associated with the development of chronic pancreatitis [81–83, 85], which suggests that *HBV*-related pancreatitis might be a precursor of PDAC [85]. Clinical observations concerning impaired pancreatic exocrine function in patients with chronic *HBV* infection support this hypothesis. The positive correlation of PDAC risk with *HBV* infection, especially for long-lasting persistent infection, chronic/inactive HBsAg carriers and occult infection is supported by unanimous conclusions drawn from meta-analyses [96–104]. Most prominently, *HBV* is capable of replicating apart from infecting in the tumor and nontumorous pancreatic tissues of PDAC patients [85]. According to the REVEAL-HBV study [105], the association between *HBV* and PDAC was found in patients with higher viral DNA loads (*HBV* DNA > 300 copies/mL). The integration of *HBV* DNA to pancreatic tissue and pancreatic metastases to the liver in patients with *HBV* infection have been confirmed [83]. Wei et al. [106] discovered that *HBV* infection in PDAC patients increased the rate of synchronous liver metastasis and this kind of status could be regarded as an independent prognostic factor. However, some studies have drawn conflicting conclusions concerning the association between the presence of *HBV*, *HCV* and PDAC [105, 107, 108].

The pancreas and liver share similar features in early embryological and fetal growth and have some common regulatory pathways in PDAC and HCC [109]. Possible mechanisms of *HBV* or *HCV* contribution to carcinogenesis include inducing inflammation [85] and modifying tissue viscoelasticity [110], DNA integration in infected cells that delays host immune system clearance of *HBV/HCV*-containing cells [96], modulating the PI3K/AKT signaling pathway via the *HBV* X protein (HBX) [86]. Another key finding is that exposure to *HBV* significantly increased PDAC risk when concomitant with diabetes [85, 96, 97], high frequency of *HBV*/*HCV* infection was reported in patients with diabetes [111], and this connection was supported by strong expression of HBsAg and HBcAg in islet cells at histological level [85]. However, pancreatic cancer cells have low levels of *HBV* replication, so molecular proofs about the potential role of *HBV* in PDAC remain limited [85, 87]. Nonetheless, available literature supports the potential etiologic and oncogenic role of *HBV* infection in PDAC. Should such findings be confirmed, they may bring new insights into the etiology and therapeutics of PDAC, and remind clinicians to prevent the reactivation of *HBV* during chemotherapy in patients with *HBV i*nfection.

#### Others

Bile is a sterile hepatobiliary solution rich in lipid, and microbial colonization in the bile fluid is defined as bactibilia. In a study based on PDAC patients, Maekawa et al. [112] investigated the presence of bacteria in bile samples via genetic sequence analysis, and the result suggested that *Enterobacter* and *Enterococcus spp.* were the major microbes. Antibody levels against *Enterococcus faecalis* capsular polysaccharide (CPS) were increased in serum of PDAC and chronic pancreatitis patients compared with normal subjects, which may indicate that infection with *E. faecalis* is involved in the progression of pancreatitis-associated PDAC. *Escherichia coli (E. coli)* is the well-known gut microbe, though outnumbered in the gut approximately one thousand to one by other species. Serra et al. found that pancreas head carcinoma (PHC) is strongly and positively correlated with bactibilia while *E. coli* and *Pseudomonas* spp. were the most common microorganisms and were negatively correlated with PHC [113]. *Transfusion-transmitted virus (TTV*) is considered hepatotropic and a possible cause of acute hepatitis. However, it has also been identified in the pancreas. Clinicians reported a case with both pancreatic cancer and *TTV* infection [114], which indicates a need for further research.

### Intrapancreatic microbiota

The pancreas was traditionally considered a sterile organ, and it has long been hold that most microbes cannot survive in pancreatic juice, which contains numerous proteases and is highly alkaline [112]. Nevertheless, compared with normal pancreatic tissue, a 1000-fold increase of bacteria in intrapancreatic was identified in PDAC patients using 16S rRNA fluorescent probes and qPCR [60, 115]. The mean relative proportions of some taxa differed among PDAC, pancreatic benign neoplasm and healthy cohort [116]. Moreover, compared with the intestinal microflora, some bacteria showed a differential increase in the pancreas of PDAC patients. Microbiota analysis using a larger cohort will be needed to make a definitive conclusion regarding the significantly distinctions in microbiome characteristics between benign and malignant pancreatic disease respectively, which may lead to the establishment of prediction markers in early diagnosis, treatment efficacy or prognosis in PDAC.

Gemcitabine has been used for advanced pancreatic cancer and was helpful in some patients, but most showed drug resistance resulting in treatment failure. Geller et al. [117] detected the presence of *Gammaproteobacteria* in PDAC tissue specimens with gemcitabine resistance and postulated that this type of bacteria could potentially modulate tumor sensitivity to gemcitabine. Pushalkar et al. [60] investigated the role of the intratumoral microbiota in PDAC progression and immunotherapy response modulation. Through a longitudinal analysis between age-matched KC (p48^Cre^; LSL-Kras^G12D^) and wild-type mice, certain bacterial populations were found to be enriched in KC mice, with the most abundant species being *Bifidobacterium pseudolongum*. These studies highlighted the significance of the intratumoral microbiota in altering the cancer natural history.

Once human pancreatic cells infected with *H. pylori,* it could colonize the pancreas and may associate with the malignant potential of adenocarcinoma [118]. A preclinical study [119] put forward that direct *H. pylori* colonization in pancreatic cancer cells, which was associated with activation of molecular pathways controlling PDAC growth and progression. However, different *Helicobacter* subspecies were identified in the pancreas and gastroduodenal tissues. Besides, *Fusobacterium* colonization in PDAC patients was identified as an independent prognostic factor for significantly shorter survival [120], in contrast to the phenomenon that oral *Fusobacterium* was associated with decreased pancreatic cancer risk.

## Potential mechanisms of microbiota roles in carcinogenesis

**Fig. 2:**
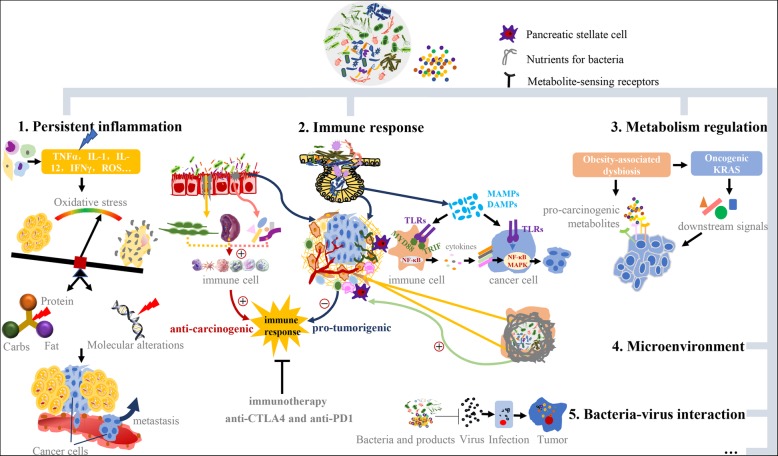
Summary of Possible Mechanisms by Which Microbiota Affect PDAC. 1) Persistent inflammation or infections acts as a central facilitator. Microbes activate inflammatory responses and ultimately lead to molecular alterations and neoplastic transformation. 2) Modulation of immune therapy in PDAC: promoting immune activation or suppression. Gut bacteria activate specific immune cells and increase their antitumor effects. Besides, the enrichment of specific strains of gut and intrapancreatic bacteria induces a tolerogenic immunosuppressive microenvironment that favors cancer progression and resistance to immunotherapies. Here, we cite an example of microorganisms within PDAC to exemplify the concrete mechanism involving TLRs, MyD88, TRIF, NF-κB and MAPK. 3) The gut microbiota serves as a critical regulator of metabolism in PDAC carcinogenesis, and obesity-associated dysbiosis is a representative pattern. 4) The microbiota is a component of the PDAC tumor microenvironment and may interact with PSCs. 5) The development of virus-associated cancers and provide a model of bacteria-virus interaction for carcinogenesis

Although the emerging preclinical data strongly support that the microbiota can influence tumor progression and therapeutic responses systemically through several pathways, including inflammation, immunity, metabolism, hormonal homeostasis, etc. [121], the molecular basis of this regulation is still being elucidated. In this section, we discuss the association between representative microbiota and PDAC as revealed in current studies. We primarily focus on exploring (i) how microbes, especially bacteria, influence carcinogenesis by perpetuating cancer-associated inflammation; (ii) the dual effect of promoting immune suppression or activation then engendering the protumorigenic effect or modulating immunotherapeutic response; (iii) the close relationship of microbes to metabolic regulation; and (iv) the microbiota as a component of the PDAC tumor microenvironment (Fig. 2). Other reported mechanisms including bacteria-virus interaction for carcinogenesis. A significant synergistic effect between microbiota composition and PDAC risk factors was also emphasized in current studies.

### Persistent inflammation or infection: a central facilitator

Inflammation is a protective or defensive response process of tissues to harmful stimuli including pathogens, involving blood vessels, immune cells, and molecular mediators. Despite there are several proposed mechanisms in microbiota-related pancreatic carcinogenesis, inflammation is a central facilitator [121]. Inflammatory conditions represented by chronic pancreatitis is a well-recognized risk factor for PDAC development, which significantly elevates the incidence rate of PDAC than healthy populations [122]. Chronic inflammation is known to participate eminently in pancreas tumorigenesis, but it is uncertain the specific causation of local inflammation. Chronic infections are the main factor inducing inflammation and cancer. Although a growing number of scientific studies suggest an underlying infectious component of pancreatic cancer etiology [43, 85, 87, 107, 108, 121, 123]. To date, no infectious origins have been established as carcinogenic for PDAC.

The microbiota, especially gram-negative bacteria, seems to be intricately linked to cancer-related inflammation status [113]. The main mediator between inflammation and cancer is the oxidative stress imbalance caused by inflammation in normal tissue and maintained by microenvironmental inflammation in malignancies. Specifically, microbes activate inflammatory responses, increase the recruitment of proinflammatory cells and secretion of cytokines, enhance exposure to oxidative stress, alter energy dynamics, and damage DNA, ultimately leading to molecular alterations and neoplastic transformation that promote tumor growth, invasion, and metastasis. Moreover, inflammation can induce the generation of angiogenetic factors and directly accelerate the survival of cancer cells by increasing oxygen and nutrient supply to tumor tissues. Besides the effects of proinflammatory cytokines, several molecular alterations such as oncogene mutations, inactivation of tumor suppressor genes, loss of heterozygosity, chromosomal and microsatellite instability, are also participate in inflammation-mediated carcinogenesis.

Not all chronic inflammation, even if systemic, can promote carcinogenesis. The formation of solid tumors is strongly related to tumor-intrinsic inflammation sustained by the protumorigenic microenvironment [124]. The cells inside the microenvironment control tumor growth by producing autocrine, paracrine and endocrine mediators [125]. Here, we propose a mechanistic framework in which microbes exert an indirect impact on tumor progression and the microenvironment [18], a direct impact on tumor initiation, and interactions with other known risk factors in pancreatic carcinogenesis. The most significant mechanistic pathways, whether extrinsic or intrinsic, between inflammation and cancer, may depend on tumor type, or perhaps both are essential [125]. The latter could be exemplified by two factors -- pancreatitis and *K-ras* gene mutations that frequently found in PDAC, and both are imperative to cause pancreatic intraepithelial neoplasia (PanIN) and invasive carcinoma in animal model [126]. That is, the mutual and combined effect of pancreatitis and RAS-RAF activation pathway [127] can induce PDAC carcinogenesis.

It is important to note that the inflammatory reaction is accompanied with the immune response, but the immune response is not necessarily to inflammation. Systemic inflammation, caused by the release of pro-inflammatory cytokines and activation of the innate immune system, might, therefore, be the accelerator and ultimate factor contributing to the development of PDAC. The interplay between the microbiota and obesity induces low-grade systemic inflammation and promotes tumor development. It has been shown that high fat/high energy diets could facilitate absorption of bacterial LPS in our guts and leading to systemic inflammation through the specific host response of TLR4 [58, 128]. Above studies support that microbes may affect pathogenesis or carcinogenesis at distant sites through systemic effects within the human body, which is more pervasively influential than our imagination.

### Modulation of immune therapy in PDAC: promoting immune activation or suppression

All risk factors are contributing to PDAC act in part via the immune response. Preliminary findings in mice and human revealed that the intestinal or intratumoral microbes can affect responses to chemotherapy via immunity and seems to influence cancer drug function. Cutting-edge immunotherapy treatment is associated with gut microbes, and microbes play a key role in innate and adaptive immune responses, maintaining the delicate balance. Also, the composition of the gut microbiome has enabled stratification of patients into responders and non-responders, thereby allowing the use of microbiota composition as a predictive biomarker of response to immunotherapy. Fecal microbiota transplant and microbe-based pills are ready for testing to determine whether they can beneficially reshape the gut microbiota of non-responders. However, a complex interplay exists among the gut microbiota, immune cells, and pancreas in pancreatic carcinogenesis, and how microbes interact with immunotherapeutics and their precise mechanisms, including which microbes modulate which immune cells, remain unsolved. In general, the relationship between microbes and immunity in PDAC carcinogenesis could be described as dual action: cancer immunity can be boosted by the microbiota, or the microbiota can exert a protumorigenic effect.

#### Cancer immunity boosted by the microbiota

Some cancer treatments rely on activating the immune system via the gut microbiota [129, 130]. The efficacy of therapies, including alkylating agents, immune checkpoint blockers and adoptive T-cell transfer (ACT), depends on immunity closely related to gut microbiota [131]. Zitvogel [132, 133] found that the chemotherapy drug cyclophosphamide damages the intestinal mucus layer, allowing some gut bacteria enter the lymph nodes and spleen, then specific immune cells being activated. In addition, cyclophosphamide lost its anticancer effects in mice when raised without microbes in guts or given antibiotics. Another study showed similar finding with oxaliplatin and cisplatin and found that the scarce of gut microbes compromises the efficacy of CpG- and anti-IL-10-based antitumor response due to ineffective priming of tumor-infiltrating myeloid cells and a consequent lack of ROS-dependent apoptosis and TNF-dependent necrosis [129].

Scientists are examining how the gut microbiota interplay with immunotherapies response and how these interactions are administrated. Following the observation that only 20~40% of patients respond to immunotherapy, Zitvogel made further efforts to explore whether gut bacteria might influence the response to anti-CTLA-4 and anti-PD-1. Their work found that microbe-free mice failed to respond to one such drug, and mouse response improved when given *Bacteroides fragilis* [134]. Sivan et al. [135] reported that *Bifidobacterium* increased cancer immunotherapy response in mice, which suggested that microbes, especially gut bacteria, might be activating the immune response by stimulating enterocytes to generate certain message molecules or provide signals to immune cells that help to supercharge their tumor-fighting efforts. Similar latest studies [136, 137] have linked favorable immunotherapy responses in melanoma patients to specific gut microbes.

Routy and colleagues [138] reported that changes in the gut microbiota composition following antibiotic usage decreased responses to immunotherapy in patients with lung, bladder, and kidney cancer. In contrast, when using antibiotics specific to certain gut bacteria in an HCC mouse model, the proportion of NK T cells in the liver increased, leading to tumor shrinkage [15]. In PDAC, the combination of antibiotics and PD-1 blockade showed that a synergistic antitumoral effect associated with T-cell activation. These confounding results suggest that each cancer type may induce a distinct profile of alterations in gut and tumor microbiota composition that may either attenuate or facilitate the function of immune checkpoint inhibitors.

#### Cancer immunity thwarted by the microbiota

The protumorigenic effect of the gut microbiota appears linked with its capacity to exert influence on immune response through the tumor microenvironment (TME), making it more tolerant toward cancer. Immune tolerance mechanisms have been implicated as the main barrier to effective antitumor immunotherapy. Pushalkar [60] showed that specific microbes in the gut and intrapancreatic serve as helpers in the establishment of immunosuppressive PDAC tumor microenvironment in spontaneous murine models, enhancing cancer progression and resistance to immunotherapies. Bacterial ablation in models showed antitumor effects and can be reversed or abrogated by the transfer of feces from PDAC-bearing KPC mice, but no difference was found when the transfer came from non-PDAC controls. These experimental data thus provide compelling preclinical evidence for gut microbiota modulation, and the tumor microbiota could sensitize the immune-refractory cancer and convert it into a more responsive one. Because bacterial ablation upregulated PD-1 expression, a clinical trial of antibiotic synergism with checkpoint-based immunotherapy is beginning, using antibiotics and pembrolizumab prior to resection in patients with locally advanced PDAC. These ideas have started to spread and create new possibilities for clear therapeutic applications of microbiota science.

Mechanistically, microorganisms in PDAC differentially activate selective TLRs in monocytes and then produce immune tolerance. TLRs, representing the most acknowledged family of pattern-recognition receptors (PRRs), are a group of pathogen-associated molecular pattern receptors and undertake a certain role in immune response to microbial infection and accelerate tumorigenesis via innate and adaptive immune suppression in PDAC. PRRs reside in most immune cells and can bind a range of microbe-associated molecular patterns (MAMPs, such as LPS), as well as byproducts of dead cells and sterile inflammation called DAMPs (damage-associated molecular patterns) [139]. After binding, the TLRs-DAMPs complex recruit MyD88 or TRIF adaptor molecules as signal transducers to activate signal pathways such as NF-κB and MAPK. The procarcinogenic effects of TLRs can be reversed by inhibiting NF-κB or MAPK pathway [140]. Miller et al. found that TLR4 and TLR7 showed up-regulated expression in the PDAC microenvironment [140, 141], and TLR signaling, such as TLR4/MyD88 [140, 142, 143], plays an important role in pancreatic tumors. Further, animal studies demonstrated that the activation of TLRs could provoke pancreatitis and synergize with *K-ras* to significantly promotes pancreatic carcinogenesis [140, 141, 144–146].

Gut microbiota ablation with antibiotics does affect the immune phenotype in the TME, that is, immunogenic reprogramming or reshaping, and then suppress tumor growth by inducing antitumorigenic T-cell activation, boosting immune surveillance and improving sensitivity to immunotherapy in malignances [147]. The immunogenic reprogramming of TME in a murine model manifests a decrease in myeloid-derived suppressor cells (MDSCs), an increase in M1 macrophage polarization, facilitating the T helper 1 (Th1) differentiation of CD4+ T cells and the activation of CD8+ T cells. All changes favor antitumor efficacy. A balance exists between pro- and antitumor T cells in tumor microenvironment. The Th1-type cytokine interferon-γ exhibits antitumorigenic effects, whereas the T helper 2 (Th2)-type cytokines interleukin (IL) 4, IL5, and IL10 and Th17 cells play a protumorigenic role. Specifically, after gut microbiota depletion in a pancreatic cancer murine model, interferon-γ producing T cells Th1 manifest with a significant increase and a corresponding decrease in IL17a and IL10-producing T cells [61]. This result is in accordance with the previous conclusion, and a high Th1/Th2 ratio in TME relates with improved survival in PDAC patients [148].

The immunologic mechanisms that underlie variable responses to systemic therapy upon changes in the microbiota were elucidated in other studies. Certain miRNAs are key regulators of the innate and adaptive immune response [52, 149, 150], and these immune-related miRNAs are secreted by cells and then travel to the pancreatic tissue to alter gene expression. They can modulate host responses to pathogens, and vice versa, pathogens also regulate miRNA expression.

### Metabolism regulation: a close association

Apparently, microbes act as a critical regulator in metabolism regulation. The effect of metabolites produced by gut microbiota on intestinal and systemic homeostasis has been verified by a substantial amount of studies [151]. Microbial metabolites do play important roles in diverse biologic and pathologic processes, including translation, gene regulation, stress resistance, and cell proliferation, differentiation, apoptosis, tumor development and aggressiveness, which were illustrated in colorectal [152–155], breast cancer [156]. As a risk factor for various diseases, obesity do result in severe social and psychological consequences, and obesity-linked gut microbiota dysbiosis exerts influence on obesity-related cancer [157, 158], including PDAC. Lower bacterial diversity and altered expression of bacterial genes are regarded as principle factors in the pathogenesis of obesity [159]. Although studies about obesity, the gut microbiota, and PDAC are rare, mechanisms have been formulated in other studies, including that abnormal microbial metabolism contributes to the production of pro-carcinogenic metabolites. DIO (diet-induced obesity) alters the gut microbiota by increasing generation of deoxycholic acid (DCA), a gut bacterial metabolite known to cause DNA damage [160]. The possibility that metabolites generated by gut microbes link dysbiosis to PDAC progression via metabolite-sensing receptors acting in pancreatic cells or other cells, merits further experiment and exploration [161].

Oncogenic *K-ras* accompanied by activation downstream effectors is insufficient in the formation process of invasive PDAC. Environmental or extrinsic factors including inflammation, metabolic, nutritional, or additional genetic mutations are also required. Changes in the gut microbiota are one of the factors “upstream” of *K-ras* linked with obesity that can enhance or modulate downstream signals [161]. Proofs in other cancer models have confirmed that dysbiosis induced by high-fat diet accelerated *K-ras*-driven intestinal tumorigenesis [128].

Surprisingly, microbiota within tumors could confer gemcitabine resistance in patients with PDAC. Cytidine deaminase (CDD) is an enzyme responsible for maintaining the cellular pyrimidine pool, which is involved in nucleic acid metabolism. Notably, certain microbes that frequently express CDD are capable of converting gemcitabine (2′,2′-difluorodeoxycytidine) into its inactive metabolite 2′,2′-difluorodeoxyuridine. Here are some examples: Geller and colleagues [117] used murine model and 113 human tissue samples of PDAC, showing that the presence of intratumoral *Gammaproteobacteria* class was responsible for inducing resistance to gemcitabine and that this effect was abolished by the use of antibiotics. When injected CDD-expressing *Escherichia coli* into the tumor-bearing mice, the researchers found that gemcitabine efficacy was dramatically impaired [162]. In addition, PDAC cells cultured with the medium that contaminated with *Mycoplasma hyorhinis* were completely resistant to gemcitabine.

### In the tumor microenvironment: further investigation needed

As previously mentioned, the microbes present within tumors located at mucosal can gradually become the composition of the tumor microenvironment in GI malignancies and affect malignant biological behavior. The TME is composed of stroma, neutrophils, macrophages, mast cells, MDSCs, dendritic cells, and natural killer cells, as well as adaptive immune cells (T and B lymphocytes) [125]. The reason that current treatments targeting PDAC cells have largely failed is that the influence of the stroma on tumor progression has been ignored. Cancer biologists focus on the cancer-cell-intrinsic mechanisms and noncancerous cells within the tumor microenvironment [163, 164] that mediate pancreatic cancer chemoresistance. Pancreatic stellate cells (PSCs) could produce the tumor stroma and play a lead role in the PDAC environment [165]. Meanwhile, microbes are a component of the PDAC tumor microenvironment [129]. Given the effect of activated PSCs on the development of PDAC microenvironment and subsequent tumor progression, and no published research to date has considered whether bacterial infections in the pancreas or other sites play a role in the activation of PSCs [166], it will be instructive and meaningful to conduct related studies.

## Conclusion

Microbiota research provides opportunities to better reveal the underlying mechanisms and identify biomarkers for predicting subsequent PDAC risk and prognosis. Previous results indicate that PDAC is associated with microbes that can potentially modulate tumor sensitivity to therapeutic agents, which is highly beneficial to improve treatment efficacy of this fatal disease via proper manipulation. Novel probiotics could be developed and used in combination with chemo and immuno-therapy, which may hold great promise for PDAC patients. Disputes exist in the microbiota field, as the microbiota is influenced by many known factors associated with PDAC risks, such as diabetes, smoking, obesity and dietary intake, and these factors impact the immune system, humoral response, and inflammation and may contribute to opportunistic infections [18, 122, 167, 168]. Collectively, it’s a pivotal time for figuring out whether the microbiota acts as a mediator of other stimuli that favoring cancer initiation and progression or itself instigates this cascade [36, 42].

To apply the microbiome in the future, we could monitor the shifts in taxon dominance during PDAC progression and develop methods targeting the cancer-associated microbiome to improve the efficacy of therapy. In particular, the debut of intestinal metagenomics and immunogenomics will help scientists achieve the additional breakthroughs in the application of the microbiota to immune therapy. However, prudent and appropriate clinical trials related to the microbiota must be performed for suitable periods (representative clinical trials of microbiota-linked for cancer are listed in Table 1). Changing the composition of microbiota might make individuals more likely to suffer from other health problems. In summary, depicting a distinct microbial landscape of PDAC is imperative, and novel approaches or strict standards are required to actively change the status quo of this field. These efforts should generate exciting clinical applications, such as development of companion diagnostics or prediction of therapeutic response, or better yet, establishing personalized medicine for each patient based on respective microbiome of themselves.

**Table 1:** Representative clinical trials of microbiota-linked for cancer

Category	Clinical Trial ID	Therapeutic Agents/Intervention	Study Phase	Status	Treatment Setting	Primary Outcomes
Oral Microbiome and Pancreatic Cancer	NCT03302637	Specimen Collection	Observational	Completed	Pancreatic Cancer	Compare bacterial taxa
Treatment of Patients with Cancer with Genetically Modified Salmonella Typhimurium Bacteria	NCT00004988	VNP20009	I	Completed	Various Types	Anti-tumor effect; maximum tolerated dose
Mixed Bacteria Vaccine (MBV) in Patients with Tumors Expressing NY-ESO-1 Antigen	NCT00623831	Bacterial Vaccine	I	Completed	Various Types	Safety; pyrogenicity; immune responses; overall response
Probiotics in Colorectal Cancer Patients	NCT00936572	Probiotics	II	Completed	Colorectal Cancer	Efficacy; immune response
Using Probiotics to Reactivate Tumor Suppressor Genes in Colon Cancer	NCT03072641	Probiotics	NA	Completed	Colon Cancer	Microbiota composition and epigenetic changes
Safety & Immunogenicity of JNJ-64041809, a Live Attenuated Double-deleted Listeria Immunotherapy, in Participants with mCRPC	NCT02625857	JNJ-64041809	I	Completed	Prostate Cancer	Safety and toxicity; ORR; PFS
Safety & Immunogenicity of JNJ-64041757, Live-attenuated Double-deleted Listeria Immunotherapy, in Subjects With NSCLC	NCT02592967	JNJ-64041757	I	Active, not recruiting	NSCLC	Safety and toxicity; ORR; PFS
Effects of Chemotherapy on Intestinal Bacteria in Patients with Newly Diagnosed Breast Cancer	NCT02370277	Specimen Collection	Observational	Active, not recruiting	Breast Cancer	Change in intestinal microbiota
Phase I/II Study of APS001F With Flucytosine and Maltose in Solid Tumor	NCT01562626	APS001F	I/II	Recruiting	Solid Tumor	Safety and toxicity
Pembrolizumab With Intratumoral Injection of Clostridium Novyi-NT	NCT03435952	Pembrolizumab Clostridium NovyiNT	I	Recruiting	Solid Tumor	Maximum tolerated dose; overall response
Engineering Gut Microbiome to Target Breast Cancer	NCT03358511	Probiotics	NA	Recruiting	Breast Cancer	Mean number of CD8+ cells
Gut Microbiome & Gastrointestinal Toxicities as Determinants of Response to Neoadjuvant Chemo for Advanced Breast Cancer	NCT02696759	Specimen Collection	NA	Recruiting	Breast Cancer	pCR
Microbiome in Lung Cancer and Other Malignancies	NCT03688347	Specimen Collection	Observational	Recruiting	Lung Cancer	Compare bacterial taxa
MRx0518 and Pembrolizumab Combination Study	NCT03637803	MRx0518 Pembrolizumab	I	Not yet recruiting	Solid Tumor	Safety and tolerability; anti-tumor effect

## Abbreviations

16S rRNA: 16S ribosomal RNA
ACT: Adoptive T-cell transfer
Cag-A: Cytotoxin-associated gene A
CDD: Cytidine deaminase
CPS: Capsular polysaccharide
DAMPs: Damage-associated molecular patterns
DCA: Deoxycholic acid
DIO: Diet-induced obesity
*E. coli*: *Escherichia coli*
ELISA: Microarray and enzyme-linked immunosorbent assay
GI: Gastrointestinal
*H. pylori*: *Helicobacter pylori*
*HBV*: *Hepatitis B virus*
HBX: HBV X protein
HCC: Hepatocellular carcinoma
LPS: Lipopolysaccharide
MAMPs: Microbe-associated molecular patterns
MDSCs: Myeloid-derived suppressor cells
*N. elongate*: *Neisseria elongate*
*P. gingivalis*: *Porphyromonas gingivalis*
PAD: Peptidyl-arginine deiminase
PDAC: Pancreatic ductal adenocarcinoma
PRRs: Pattern-recognition receptors
PSCs: Pancreatic stellate cells
qPCR: quantitative polymerase chain reaction
ROS: Reactive oxygen species
*S. mitis*: *Streptococcus mitis*
Th1: T helper 1
Th2: T helper 2
TLRs: Toll-like receptors
TME: Tumor microenvironment
